# Application of biodegradable collagen matrix (Ologen™) implants in Dacryocystorhinostomy surgeries, a randomized clinical study

**DOI:** 10.1186/s12886-018-0901-4

**Published:** 2018-09-20

**Authors:** Hatem M. Marey, Hesham M. Elmazar, Sameh S. Mandour, Osama A. El Morsy

**Affiliations:** Department of Ophthalmology, Menoufia Faculty of Medicine, Shebin El Kom, Menoufia, Egypt

**Keywords:** Acquired nasolacrimal duct obstruction (NLDO), Dacryocystorhinostomy (DCR), Ologen implants

## Abstract

**Background:**

To introduce and evaluate the application of Ologen implants in external Dacryocystorhinostomy (DCR) Surgeries.

**Methods:**

Prospective comparative randomized study was carried out on 60 patients coming to ophthalmology department, Menoufia University Hospitals. Patients included were suffering from primary acquired nasolacrimal duct obstruction with positive regurge test. Patients were randomly enrolled into two groups using alternating choice technique. Group A included 30 patients who had DCR surgery to treat the obstruction with Silicone tubes. Group B included 30 patients had a Dacryocystorhinostomy with Silicone tubes and Ologen implants.

**Results:**

Success rates as regard to relief of symptomatic epiphora were 86.7% in group A and 96.7% in group B and time of dye clearance test was 4.5 ± 0.6 min in group A and 3.9 ± 0.4 min in group B with *p* value 0.353 &0.001 consecutively. Apart from immediate mild post operative hemorrhage that was encountered in 2 cases in group B and 1 case in group A, there were no significant complications in both groups.

**Conclusion:**

The current study shows that application of Ologen implants in external DCR surgeries may improve symptomatic epiphora without exposing the patients to more intra-operative or post-operative complications. To the best of our knowledge, the current study is the first one to use Ologen implants in external DCR surgeries. However, the follow-up period was relatively short and the sample size is relatively small and further work is required to verify the effect of Ologen in external DCR surgeries.

**Trial registration:**

Current Controlled Trials PACTR201711002809215, and the date of registration is 29 November 2017. The trial is Retrospectively registered.

## Background

Dacryocystorhinostomy (DCR) is a procedure that creates an epithelium-lined tract between lacrimal sac and nasal mucosa bypassing the occluded nasolacrimal duct. The classic procedure has passed through many minor modifications; however, the basic technique still has a high success rate [1, 2]. A Silicone tube is used to keep the created fistula open specially if anterior flaps are not adequately demarcated and separated from posterior flaps. Recurrence of symptoms may result from closure of the new tract due to excess fibrosis at the osteotomy site [3].

The Ologen™ is a relatively new material composed of lyophilized porcine atelocollagen (> 90%) and lyophilized porcine glycosaminoglycan (< 10%) with pore sizes of 10 to 300 μm. It provides a biodegradable, implantable scaffold collagen matrix. Atelocollagen (the main component) is a highly purified pepsin-treated type I collagen which has low immunogenicity, because it is free of telopeptides [4]. A telopeptide is an amino acid sequence at both N and C terminals, which is responsible for most of the collagen’s antigenicity. The porous structure of Ologen™ directs fibroblasts and myoblasts to form a loose connective tissue minimizing scar formation.

Ologen has been tried as a conjunctival autograft for scleral necrosis after pterygium excision with a good results [5], and to control conjunctival fibrosis in subscleral trabeculectomy [6].

The aim of this study is to investigate the role of Ologen implants in regulating the healing process after external DCR surgery to prevent early fibrosis.

## Methods

This is a prospective comparative randomized study. It was carried out at Ophthalmology Department, Menoufia University Hospitals, Egypt, in the period from Jan 2013 till Feb 2016. The study included 60 patients suffering from acquired nasolacrimal duct obstruction with positive regurgitation test. They were randomly enrolled into two groups. Group A included 30 patients who underwent DCR with Silicone tubes, and group B included 30 patients who underwent DCR with Silicone tubes and Ologen implants. Patients were allocated into either group using alternating choice technique with respect to equal match for both age and sex.

Patients included in the study were suffering from primary acquired naso lacrimal duct obstruction (NLDO) with positive regurgitation test and delayed dye clearance test more than 10 min in all cases without any previous surgery. Patients with any secondary causes for NLDO, negative regurgitation test, or history of previous surgery were excluded from the study.

After obtaining the necessary approval from the Faculty Ethics Committee of Faculty of Medicine, Menoufia University, a detailed discussion about the risk and benefits of the operation was carried out with all patients and a written consent was obtained. All measures were in accordance with the tenets of the Declaration of Helsinki.

All patients included in the study had detailed ophthalmologic examination including visual acuity, detailed eyelid examination and regurgitation test. Nasal examination was carried out by an Ear, Nose and Throat specialist to rule out nasal causes for obstruction of nasolacrimal duct (NLD) opening in the inferior meatus as deviated septum or nasal polyps. CT was done for all cases to assess the nasal cavity, anatomy of the bony lacrimal fossa and if there is anterior extension of ethmoid air cells into bony lacrimal fossa as well as to exclude malignant NLD obstruction.

### Surgical technique

Patients of both groups were operated under general anesthesia with naso-tracheal intubation and packing of the oro-pharyngeal area. . All surgeries were done by the second and third authors (HFE & SSM).

**Fig. 1:**
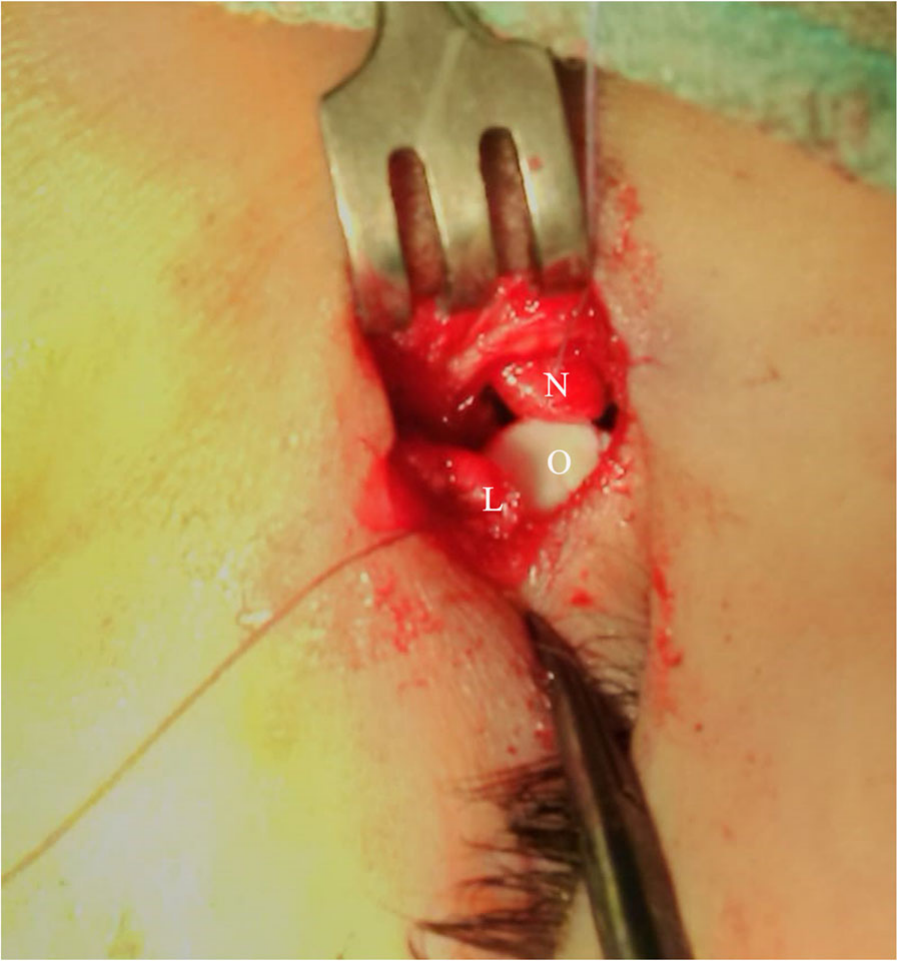
Ologen implant (O) in position under the nasal mucosal flap (N) and the lacrimal sac flap (L) with stitches passing through the implant for fixation

A vertical small 10 mm incision line was marked on the side of the nose 11 mm from the medial canthus. The bone of the anterior lacrimal crest and the lacrimal fossa was exposed to create an adequate osteotomy to anastomose the sac and nasal mucosa. The posterior flaps of lacrimal and nasal mucosa were cut. Silicone tube was inserted and threaded through the puncti in the lacrimal passages till it appeared in the opened lacrimal sac (Fig. 1). Then, it was delivered through the nasal cavity.

In group B, Ologen™ collagen matrix (OCM, Aeon Astron Europe B.V., Leiden, and The Netherlands) 1 cm × 1 cm was placed under the anterior flaps of both the lacrimal sac and nasal mucosa . 6/0 Vicryl suture was then passed from the anterior nasal mucosal flap, through the Ologen implant underneath, to the anterior lacrimal sac flap . The suture was returned back in the opposite direction and tied over the anterior nasal mucosal flap in a mattress fashion.

In both groups, skin and muscles were sutured with 6/0 Vicryl sutures. Nasal packing was not necessary unless profuse bleeding occurs. Postoperative treatment with antibiotics for 1 week was scheduled in all cases. Silicone tubes were left in place for 6–12 weeks. Follow up period extended up to 18 months.

Post operative visits were scheduled after 1 week, 1 month, 3 months and 6 monthly thereafter. Detailed ophthalmic examination was done in these visits to evaluate patent satisfaction and absence of preoperative symptoms. Post operative complications were detected, documented and properly managed. We stressed on following up the level of tear meniscus by slit lamp examination, dye clearance test, regurgitation test and patient satisfaction. Success in our study was defined as complete absence of epiphora or occasional epiphora requiring drying less than 2 times per day, 80–100% improvement in the timing of preoperative dye clearance test, negative regurgitation test, and the patient is satisfied, otherwise, it was considered as failure.

Data were collected, tabulated and analyzed by SPSS. We used t-test to compare the age, and the follow up period in Table 1, and dye clearance test in Table 3, chi square test to compare gender in Table 1, and Fischer exact test to compare hemorrhage, wound healing problems, and hypertrophic scar in Table 2, and presence of epiphora in Table 3.

**Table 1:** Descriptive data for both groups

Variable	Group A	Group B	*p* value	Test
Age				
Mean ± SD	51.27 ± 5.42	50.53 ± 5.64	*p* = 0.719	t test
Gender				
Male	6	8	*p* = 0.093	Qui Square test
Female	24	22		
Follow up period in months	14.87 ± 2.1	15.13 ± 1.85	*p* = 0.722	t test

**Table 2:** Post-operative data for both groups

Variable	Group A	Group B	*p* value	Test
Hemorrhage				
Present	1	2	*p* = 1	Fischer exact test
Absent	29	28		
Wound healing problems				
Present	2	1	*p* = 1	Fischer exact test
Absent	28	29		
Hypertrophic scar				
Present	1	1	*p* = 1	Fischer exact test
Absent	29	29

**Table 3:** Success rate, dye clearance test for both groups

Variable	Group A	Group B	*p* value	Test
Epiphora				
Present	4	1	*p* = 0.353	Fischer exact test
Absent	26	29		
Dye Clearance Test (minutes)	4.5 ± 0.6	3.9 ± 0.4	*p* = 0.001	t test

### Results

The study included 60 patients divided into 2 equal groups, A; included 30 cases (as a control group) and B; included 30 cases (as a case group). The mean age in group A was 51.27 ± 5.42 years and in group B was 50.53 ± 5.64 years. Females were more than males in both groups with a female: male ratio 4 to1 in group A and 11 to 4 in Group B. The follow up period extended for 12 up to 18 months with a mean of 14.87 ± 2.1 months and 15.13 ± 1.85 months for group 1 and 2 respectively. There was no statistically significant difference between the 2 groups regarding age, sex, and follow up period as shown in Table 1.

Follow up data included; post-operative hemorrhage, wound healing problems, hypertrophic cutaneous scar, dye clearance test, and postoperative epiphora.

There was no statistically significant difference regarding problems of wound healing and incidence of postoperative hypertrophic cutaneous scars. As well, there was no statistically significant difference regarding post-operative hemorrhage within first 24 h. It was encountered in 1 case in group A and in 2 cases in group B, a problem that was addressed by nasal packing and prompt control of arterial blood pressure. As shown in Table 2.

At the end of the follow up period, success rates for relief of symptomatic epiphora was (26 cases) 86.7% in group A, and (29 cases) 96.7% in group B. with *p* value of 0.353. The time of dye clearance test has improved to 4.5 ± 0.6 min in group A and 3.9 ± 0.4 min in group B with *p* value 0.001, as shown in Table 3.

## Discussion

External DCR is an effective procedure for managing epiphora caused by nasolacrimal duct obstruction. Its success rate is higher than endnonasal DCR. “Volume” symptoms (due to fluid retention in the lacrimal sac) can be cured in every case if there are no canalicular or eyelid abnormalities. “Flow” symptoms will improve in 95% of cases as they are affected by efficiency of canalicular conductance [7–10]. Failure of DCR is multifactorial, however, excessive fibrosis at the rhinostomy site represents a major postoperative problem [11].

Mitomycin C (MMC) has been used in lacrimal drainage surgery. A meta-analysis found that intraoperative MMC application was a safe adjuvant that could reduce the rate of closure of the rhinostomy site after primary external DCR [12]. Other controlled studies have investigated adjunctive MMC for primary or revised endoscopic DCR to augment the surgical success rate. However, the results are not completely consistent [13–15].

In the current study, Ologen™ was expected to have an effect similar to what happen when it is used in conjunctival surgeries. The porous structure of Ologen™ (during the period of its existence which extends for 90–180 days before complete degradation) directs fibroblasts and myoblasts to form a loose connective tissue matrix during the process of wound healing which is remarkably similar to normal tissue, with less scar formation [16].

Ologen™ was used successfully to control conjunctival fibrosis in subscleral trabeculectomy in previous report [6]. Nasal mucosa is one of the highly vascular structures in the body. Hence, high incidence of fibrosis following DCR was encountered In the current study, authors tried to minimize the postoperative fibrosis of nasal mucosa by using a fibro vascular regulating substances like Ologen™. There was improvement in postoperative epiphora in the study group (96.7% success rate) as compared to control group (86.7% success rate). However, the difference between the two groups was not statistically significant and this may be attributed to the small sample size in the current study.

Wound healing problems were recorded in 2 cases in group A and 1 case in group B. As well, hypertrophic cutaneous scar was recorded in 1 case from each group. However, the hypertrophic scar was improved during follow up period. This is in agreement with Walland et al. who noticed improvements in the cosmetics of the external DCR scar with time to be almost imperceptible by 6 months postoperatively [17].

We encountered post-operative hemorrhage within first 24 h in 1 case in group A and in 2 cases in group B. This may result from the presence of Ologen implant which keeps the ostium patent and delay fibrosis. Postoperative bleeding was described in a low rate in classic DCR surgeries and could be managed with postoperative lowering of the blood pressure to normal values and by nasal packing [3].

## Conclusion

To the best of our knowledge, the current study is the first to investigate the role of Ologen implantation in reducing fibrosis in external DCR. It was found to be a potentially safe and easy technique that helps improving the surgical outcomes without intra operative or post operative complications. Although, the *P*-value was not significant (0.3) between the non-Ologen and Ologen group, there was a difference in clinical improvement (86% vs 96% respectively). The statistically non-significant difference may be attributed to small sample size. Therefore, further work is required to verify Ologen effect on a longer follow up period and on a larger scale of cases. As well, further studies may investigate the application of Ologen without tubes which if found effective will reduce the time of surgery and eliminate the need for another intervention to remove the tubes.
